# Misleading HbA1c Measurement in Diabetic Patients with Hemoglobin Variants

**DOI:** 10.3390/medsci9020043

**Published:** 2021-06-07

**Authors:** Manthana Mitchai, Nattakarn Suwansaksri, Suphakdee Seanseeha, Jindamanee Saenboonsiri, Putthichai Kraitree, Jirasak Piyapromdee, Atit Silsirivanit

**Affiliations:** 1 Medical Molecular Biology Center, Department of Clinical Pathology, Khon Kaen Hospital, Ministry of Public Health, Khon Kaen 40000, Thailand; suphakkku@yahoo.com (S.S.); sebonii@yahoo.com (J.S.); putthichai3608@gmail.com (P.K.); 2 Department of Internal Medicine, Khon Kaen Hospital, Ministry of Public Health, Khon Kaen 40000, Thailand; natta.suwan@gmail.com (N.S.); jirasak21@gmail.com (J.P.); 3 Department of Biochemistry, and Center for Translational Medicine, Faculty of Medicine, Khon Kaen University, Khon Kaen 40002, Thailand; atitsil@kku.ac.th

**Keywords:** capillary electrophoresis, glycated hemoglobin, hemoglobin A1c, hemoglobin variant

## Abstract

Background and Objectives: Hemoglobin A1c (HbA1c) is widely used for the monitoring and management of diabetes mellitus. The aim of this study is to investigate the influence of hemoglobin (Hb) variants on the measurement of HbA1c. Materials and Methods: HbA1c levels of 845 blood samples obtained from diabetic patients with various hemoglobin types were measured using a turbidimetric inhibition immunoassay and capillary electrophoresis. Results: Of 845 patients with diabetes, 65.7% (555/845) have the normal hemoglobin type (A_2_A) and 34.3% (290/845) have various abnormal hemoglobin types, including heterozygous HbE 30.2% (255/845), homozygous HbE 1.9 % (16/845), Hb Constant Spring (CS) trait 1.4% (12/845), CSEA Bart’s 0.2% (2/845), and beta-thalassemia trait 0.6% (5/845). In most of the patients with diabetes, HbA1c levels determined by two different methods, inhibition immunoassay and capillary electrophoresis, gave strong positive correlation (*R* = 0.901, *P* < 0.001), except for those with homozygous HbE (*N* = 16) and CSEA Bart’s (*N* = 2). In all 18 patients with homozygous HbE and CSEA Bart’s, the HbA1c was undetectable by capillary electrophoresis, meaning that their estimated average glucose was undeterminable, although their HbA1c levels could be measured using an inhibition immunoassay. The discrepancy of HbA1c results obtained from two different methods is noted in patients without HbA. Conclusions: We have demonstrated the erroneous nature of HbA1c measurement in patients with hemoglobin variants, especially in those without HbA expression. Therefore, in the population with a high prevalence of hemoglobinopathies, hemoglobin typing should be considered as basic information prior to HbA1c measurement.

## 1. Introduction

Glycated hemoglobin (Hb), described as hemoglobin A1c (HbA1c), is a glycemic control marker used for the monitoring and prediction of complication risks in patients with diabetes mellitus (DM). The HbA1c level reflects an average of glucose concentrations over the most recent 2–3 months [1]. Several methods, with different limitations and advantages, are available for HbA1c measurement in clinical laboratories: boronate affinity assay, capillary electrophoresis (CE), high-performance liquid chromatography (HPLC), and turbidimetric inhibition immunoassay (TINIA) [2,3]. Many factors, e.g., blood transfusion, hemoglobin variants, hemorrhage, iron deficiency, anemia, and red blood cell half-life, are considered to affect HbA1c measurements [4,5,6]. Recent studies revealed that hemoglobinopathies can affect HbA1c measurements; for example, the Hb1Ac level was underestimated in patients with Hb variant phonotypes [7,8].

The frequency and distribution of Hb variants were variable among different areas; sickle cell anemia-related Hb (HbS) is mostly found in Africa and some areas of the Mediterranean, Middle East, and India [9]. HbE is frequently found in Bangladesh, Myanmar, East and Southeast Asia [9]. During the study of patients with diabetes, Hb variants were found in as high as one-third of all patients [8]. Thalassemia is the most common hemoglobinopathy in Thailand with varied frequencies among different areas, from 3–9% for β-thalassemia, 20–30% for α-thalassemia, and 10–60% for HbE phenotypes [10,11,12,13]. Based on the high prevalence of Hb variants among the Thai population, suggesting the possible misinterpretation of HbA1c, this study aimed to investigate the influence of Hb variants on the HbA1c measurement in patients with diabetes.

## 2. Materials and Methods

### 2.1. Blood Samples

The blood samples used were the leftover specimens from 845 patients with diabetes who visited the diabetic clinic, out-patient department, Khon Kaen Hospital, Khon Kaen, Thailand (January 2019–January 2020). The inclusion criteria were as follows: (i) age over 20 years, (ii) no blood transfusion history, and (iii) not pregnant. The study was conducted according to the guidelines of the Declaration of Helsinki, and approved by the Ethical Committee of Khon Kaen Hospital, Khon Kaen, Thailand (KE61135), which waived the requirement for informed consent.

### 2.2. Hemoglobin A1c Analyses

The HbA1c level was measured using two different methods: (i) capillary electrophoresis using a Capillarys 2 Flex Piercing CE system (Sebia, Paris, France), and (ii) turbidimetric inhibition immunoassay using a Roche Co-bas c501 automatic analyzer (Roche Diagnostics, Mannheim, Germany), according to the manufacturers’ instructions. Both systems were certified by the International Federation of Clinical Chemistry and Laboratory Medicine (IFCC) and the National Glycohemoglobin Standard Program (NGSP).

### 2.3. Hemoglobin Typing

Hemoglobin type was determined using a standard capillary electrophoresis system (Capillarys 2FP), according to the manufacturer’s instructions (Sebia).

### 2.4. Plasma Glucose and Estimated Average Glucose Analyses

Fasting blood glucose (FPG) level was determined using a glucose oxidase enzymatic assay system (Roche Diagnostics), according to the manufacturer’s instruction. The estimated average glucose (eAG) was calculated from the HbA1c level as described previously [14], using Equation (1):

eAG (mg/dL) = (28.7 × HbA1c) − 46.7 (1)

### 2.5. Statistical Analysis

Data analysis was performed using GraphPad Prism version 9.0 (GraphPad, CA, USA). Correlation between FPG and Hb1Ac was analyzed using simple linear regression and Pearson correlation analyses. The difference in HbA1c levels obtained from CE and TINIA was assessed by Bland–Altman plot. Student’s *t*-test was used to compare the difference between the groups. The *P*-value < 0.05 was considered to be statistically significant.

## 3. Results

### 3.1. Prevalence of Hemoglobin Variants among Patients with Diabetes

Hb types were determined for 845 blood samples from patients with diabetes. The results showed that 555 patients (65.7%) had normal Hb (A_2_A) and 290 patients (34.3%) had hemoglobinopathy phenotypes. The prevalence of each of the phenotypes among the total subjects was shown in Table 1; heterozygous E (*N* = 255, 30.2%), homozygous E (*N* = 16, 1.9%), Constant Spring (CS) trait (*N* = 12, 1.4%), β-thalassemia trait (*N* = 5, 0.6%), and Constant Spring EA Bart’s (CSEA Bart’s, *N* = 2, 0.2%).

### 3.2. HbA1c Levels of Patients with Hemoglobinopathies

**Figure 1:**
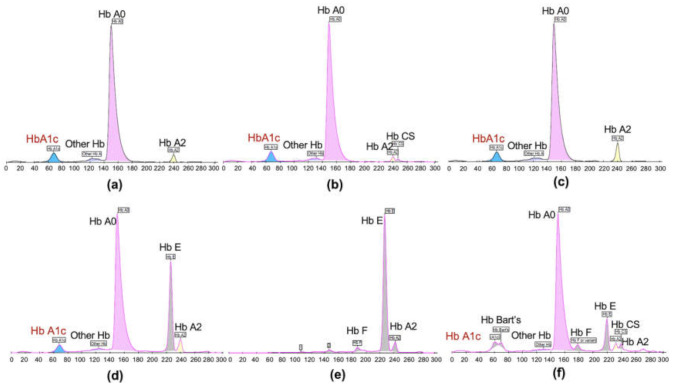
Chromatogram of HbA1c determination on CE of Hb variants’ samples. (**a**) A2A: Normal; (**b**) Hb CS trait; (**c**) β-thalassemia trait; (**d**) Heterozygous HbE; (**e**) Homozygous HbE; (**f**) CSEA Bart’s.

Hb1Ac levels of patients with diabetes were measured using two methods: CE and TINIA. Using the CE method, each type of Hb was separated based on its chemical/physical properties and appeared as a separate peak in the chromatogram (Figure 1). HbA1c was seen as an additional peak that was separated from Hb A0 (Figure 1a). Likewise, HbA1c was separated from each of the Hb types of hemoglobinopathies (Figure 1b–d). The specific peak of HbA1c was not detected in the samples from patients with homozygous HbE (*N* = 16, Figure 1e) and CSEA Bart’s (*N* = 2, Figure 1f), therefore the HbA1c levels of those measured by CE were not available for these cases.

As shown in Table 1, comparing the levels of HbA1c and FPG among the groups of patients, our results showed that HbA1c and FPG were comparable among these groups and not statistically different. HbA1c levels determined by TINIA were slightly higher than those determined by CE, although not statistically significant. In patients with homozygous HbE and CSEA Bart’s, HbA1c was not detected by CE, but it was determined as 7.67 ± 1.98% by TINIA.

### 3.3. Comparison of HbA1c Levels Measured by Two Methods

**Figure 2:**
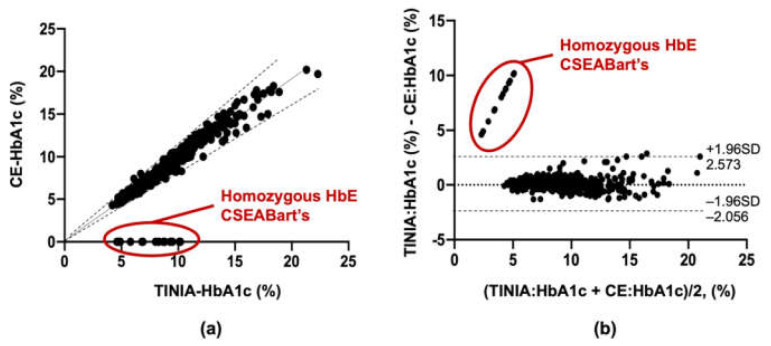
The correlation between the HbA1c level in 845 patients with diabetes measured by capillary electrophoresis (CE) and turbidimetric inhibition immunoassay (TINIA), represented by (**a**) simple linear regression plot and (**b**) Bland–Altman plot. Dotted line represented 95% CI.

Since HbA1C measurements by CE and TINIA are based on fundamentally different principals, the results obtained by those two methods were compared by correlation analysis. Simple liner regression analysis showed that, except for 18 patients with homozygous HbE and CSEA Bart’s, the HbA1c level of all of the other 825 patients measured by CE and TINIA gave a linear relationship with R-square (*R^2^*) of 0.8121 (Figure 2a) and corresponding equations: y = 0.8259x − 0.1720. Pearson correlation analysis also supported the positive correlation between HbA1c levels measured by CE and TINIA with *R* = 0.901 and a 95% confident interval (CI) of 0.888 − 0.913 (*P* < 0.001), once the data of 18 patients with homozygous HbE and CSEA Bart’s were excluded. Moreover, the results of the Bland–Altman plots were in agreement with the HbA1c level measured by these two methods with a 95% confidence interval (Cl) of −2.056 to 2.573% (Figure 2b), when the data of patients with homozygous HbE and CSEA Bart’s (red circled, Figure 2b) were excluded.

### 3.4. Effects of Hemoglobinopathy on Estimated Average Plasma Glucose and Fasting Blood Sugar

**Figure 3:**
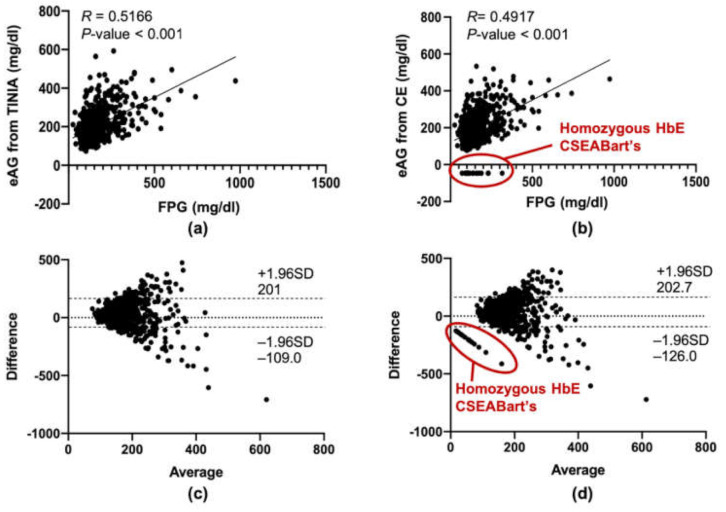
Correlation of fasting plasma glucose (FPG) and estimated average glucose (eAG) calculated from the HbA1c level obtained from TINIA and CE was analyzed using Pearson correlation (**a**,**b**) and Bland–Altman plot (**c**,**d**). X-axis is the mean between TINIA and CE (%). Y-axis is the mean between TINIA and CE (%). Dotted line represented 95% CI.

Estimated average glucose (eAG) is calculated based on HbA1c level. As shown in Figure 3, as is expected, the level of eAG either measured by CE (Figure 3a) or TINIA (Figure 3b) significantly correlated with FPG (*R* = 0.492 (95% CI = 0.440–0.542) for CE and *R* = 0.517 (95% CI = 0.466–0.564) for TINIA) (*p* < 0.001) in linear correlation analysis, when the data of patients of homozygous HbE and CSEA Bart’s were excluded (red circled, Figure 3a). Bland–Altman analyses showed that the estimated average glucose (eAG), which was calculated from HbA1c, was slightly higher than FPG with a bias of 38.31 mg/dL and 95% Cl of −126.0 to 202.7 mg/dL for those measured by CE, and a bias of 45.9 mg/dl and −109 to 201 mg/dl by TINIA (Figure 3c,d). In our findings, MCV with less than 80 fl was found to have a negative association with HbE quantity (Supplementary Materials).

## 4. Discussion

The HbA1c value reflects the patient’s mean glycemic level in the past 6–8 weeks and has been referred to as one of the major markers for diabetes diagnosis by the World Health Organization since 2011 (http://www.who.int/diabetes/publications//report-hha1c_2011.pdf, accessed date: 30th January 2021). The International Federation of Clinical Chemistry and Laboratory Medicine defined HbA1c as Hb that is irreversibly glycated at the N-terminal valine of alpha-chains. Although all commercially available methods include HbA1c in glycate hemoglobin measurements, their ability to detect non-A1c glycate hemoglobin is variable. Hemoglobinopathy alters the composition and structure of hemoglobin and leads to misinterpretation of the results of HbA1c measurements. Hemoglobin variants potentially affect the precision of current methods of HbA1c measurement [15]. Thalassemia and hemoglobinopathies are common in the Thai population; a high prevalence of variants of Hb and abnormal Hbs, including α-thalassemia, β-thalassemia, and the HbE carrier has been reported in the Northeastern region [16,17]. In this study, one-third of patients with diabetes studied had Hb variants, including heterozygous HbE (30.2%) and homozygous HbE (1.9%), which is comparable with the general population, as previously reported [17]. The result showed that, except for homozygous HbE and CSEA Bart’s, HbA1c levels of all other patients determined by two different methods were not statistically significantly different.

The eAG value is converted from the HbA1c level. Postprandial hyperglycemia largely contributes to overall HbA1c levels. In this study, we postulated that the deviation of eAG-FPG might be caused by the interference of the HbA1c measurement by variant Hbs, resulting in the discordance of FPG and HbA1c. Previous studies showed that the resolution of CE allows the separation of many common and rare Hb variants from the HbA fraction [18,19]. Our results showed that HbA1c in 16 homozygous HbE cases without HbA and two cases of CSEA Bart’s was undetectable by CE, although the HbA1c levels of all of them were available by TINIA. The result from the CE method is based on the measurement principle used to determine HbA1c by physical property and charge of Hb. The chromatography of CE precisely separates HbA1c from other Hb variants; it is therefore reasonable that HbA1c was undetectable in patients with homozygous HbE, as there is no HbA presented in this group. On the other hand, TINIA was based on the reactivity of the antibody, the N-terminal glycated amino acid of the hemoglobin beta chain. Hb variants with alterations in the first 4–10 N-terminal amino acids could produce similar results. Patients with the HbE phenotype presented HbE1c, which possibly affected the measurement of the HbA1c percentage, as the total glycated-Hb was a sum of HbA1c and HbE1c [20]. As the chromatograms of Hb Bart’s and HbA1c were extremely close and difficult to distinguish, this might be the reason why HbA1c was not reported in the two cases of CSEA Bart’s. In addition, other biological factors may affect HbA1c quantification, including shortened red cell survival or decrease of mean erythrocyte age, which are to be considered as recommended by the National Glycohemoglobin Standardization Program (NGSP). Red blood cell survival of β-thalassemia and thalassemia intermedia was shorter than normal [21]. It is critically important to identify the clinical status where there is significantly decreased red cell survival, as the HbA1c will be falsely lowered. The current reference range of HbA1c is perhaps applicable only for patients with normal Hb typing; the new reference range is suggested to be customized for those with Hb variants.

In regions where the population of Hb variants is highly prevalent, clinical laboratories in the regions should be aware of the limitations of their glycate hemoglobin determination to allow accurate results of HbA1c in these individuals. It is important to know that the Hb variants are an underlying factor of patients with diabetes before HbA1c determination. Patients with Hb variants are encouraged to use non-Hb-based methods, such as fructosamine, glycate albumin, or continuous glucose monitoring to access long term glycemic control, instead of HbA1c measurement. Furthermore, decreased MCV is an associated marker which clinicians should be investigating when interpreting HbA1c results from diabetes with Hb variants.

## Figures and Tables

**Table 1:** Hb typing and HbA1c level in diabetes patients.

Hemoglobin Typing	*N*	Prevalence (%)	FPG (mg/dL)	HbA1c (%)		
				TINIA	CE	*p*-Value
**Normal**						
A2A	555	65.7	154.0 ± 85.55	8.62 ± 2.47	8.49 ± 2.41	0.351
**Hemoglobin variants**						
Heterozygous HbE	255	30.2	156.2 ± 87.19	8.64 ± 2.75	8.57 ± 2.79	0.788
Homozygous HbE CSEA Bart’s	16 2	1.9 0.2	144.1 ± 62.93	7.67 ± 1.98	Undetectable	***-***
Hb CS trait β-thalassemia trait	12 5	1.4 0.6	152.4 ± 45.27	7.75 ± 2.89	7.81 ± 2.89	0.953

Numbers presented are mean value ± standard deviation (SD); *p*-value (T-test comparing TINIA *vs* CE); FPG: fasting plasma glucose; TINIA: turbidimetric inhibition immunoassay; CE: capillary electrophoresis

## Supplementary Materials

The following are available online at https://www.mdpi.com/article/10.3390/medsci9020043/s1: Table S1: Demographic data of patients; Table S2: Pearson correlation blood parameters in patients with diabetes.
