# Supplementary material for: Misleading HbA1c Measurement in Diabetic Patients with Hemoglobin Variants
Source: Med Sci (Basel). 2021 Jun 7;9(2):43. doi: 10.3390/medsci9020043 (PMC8293317; doi:10.3390/medsci9020043)
Supplement: Supplementary file 1 [file medsci-09-00043-s001.zip › medsci-1233849-supplementary.pdf]

Supplementary Material

Misleading HbA1c Measurement in Diabetic Patients with Hemoglobin Variants

Manthana Mitchai[1*], Nattakarn Suwansaksri[2], Suphakdee Seanseeha[1], Jindamanee Saenboonsiri[1], Putthichai Kraitree[1], Jirasak Piyapromdee[2], Atit Silsirivanit[3]

[1] Medical Molecular Biology Center, Department of Clinical Pathology, Khon Kaen Hospital, Ministry of Public Health, Khon Kaen, 40000, Thailand; (M.M.) manthanakkh@gmail.com, (S.S.) suphakkku@yahoo.com, (J.S.) sebonii@yahoo.com, (P.K.) putthichai3608@gmail.com

[2] Department of Internal Medicine, Khon Kaen Hospital, Ministry of Public Health, Khon Kaen, 40000, Thailand; (N.S.) natta.suwan@gmail.com, (J.P.) jirasak21@gmail.com

[3] Department of Biochemistry, and Center for Translational Medicine, Faculty of Medicine, Khon Kaen University, Khon Kaen, 40002, Thailand; (A.S.) atitsil@kku.ac.th

* Correspondence: manthanakkh@gmail.com; Tel.: +66-43-009900 ext.4001

Table S1. Demographic data of patients

Characteristics	Values
Sex (N)	
Male	385 (45.4%)
Female	461 (54.6%)
Age (mean± SD), years	58.6 ± 14.6
Fasting blood sugar (mean± SD), mg/dl	153.0 ± 85.8
HbA1c (mean± SD), %	
CE	8.3± 2.8
TINIA	8.6± 2.6
Hematology parameter (mean± SD)	
Red blood cell count, x10^6 cells/ul	4.4±0.9
Hemoglobin (Hb), g/dl	11.8±2.8
Hematocrit (Hct), %	35.3±6.8
Mean corpuscular volume (MCV), fl	80.6±8.6
Mean corpuscular hemoglobin (MCH), pg	27.9±3.2
Mean corpuscular hemoglobin concentration (MCHC), g/dl	33.5±1.7

Table 2. Pearson correlation blood parameters in patients with diabetes.

	A1C (TINIA)	A1C (CE)	%HbA	%HbA2	%HbE	%HbF	RBC count	Hb	Hct	MCV	MCH	MCHC
**FPG**	r= 0.517<br>p < 0.001	r = 0.493<br>p < 0.001	r = 0.001<br>p = 0.994	r = 0.017<br>p = 0.627	r = -0.037<br>p = 0.488	r = 0.172<br>p = 0.056	r = 0.040<br>p = 0.250	r = 0.007<br>p = 0.841	r = -0.011<br>p = 0.756	r = -0.062<br>p = 0.076	r = -0.002<br>p = 0.960	r = 0.119<br>p < 0.001
**A1C-Roche**	-	r = 0.905<br>p < 0.001	r = 0.023<br>p = 0.497	r = -0.007<br>p = 0.844	r = -0.050<br>p = 0.350	r = 0.176<br>p = 0.051	r = 0.193<br>p < 0.001	r = 0.112<br>p = 0.001	r = 0.121<br>p < 0.001	r = -0.117<br>p < 0.001	r = -0.093<br>p < 0.001	r = 0.028<br>p = 0.414
**A1C-CE**	-	-	r = 0.273<br>p < 0.001	r = -0.146<br>p < 0.001	r = -0.426<br>p < 001	r = 0.108<br>p = 0.236	r = 0.146<br>p < 0.001	r = 0.139<br>p < 0.001	r = 0.152<br>p < 0.001	r = 0.012<br>p = 0.738	r = 0.026<br>p = 0.459	r = 0.036<br>p = 0.305
**%HbA**	-	-	-	r = -0.359<br>p < 0.001	r = -0.993<br>p < 0.001	r = -0.072<br>p = 0.429	r = -0.172<br>p < 0.001	r = 0.101<br>p = 0.004	r = 0.117<br>p < 0.001	r = 0.508<br>p < 0.001	r = 0.454<br>p < 0.001	r = -0.021<br>p = 0.550
**%HbA2**	-	-	-	-	r = 0.766<br>p < 0.001	r = 0.115<br>p = 0.203	r = 0.065<br>p = 0.059	r = -0.050<br>p = 0.151	r = -0.067<br>p = 0.055	r = -0.225<br>p < 0.001	r = -0.197<br>p < 0.001	r = 0.024<br>p = 0.496
**%HbE**	-	-	-	-	-	r = 0.235<br>p < 0.001	r = 0.217<br>p < 0.001	r = -0.101<br>p = 0.064	r = -0.095<br>p = 0.080	r = -0.586<br>p < 0.001	r = -0.541<br>p < 0.001	r = 0.001<br>p = 0.999
**%HbF**	-	-	-	-	-	-	r = 0.005<br>p = 0.961	r = -0.138<br>p = 0.137	r = -0.124<br>p = 0.137	r = -0.212<br>p < 0.001	r = -0.226<br>p < 0.001	r = -0.047<br>p = 0.613
**RBC count**	-	-	-	-	-	-	-	r = 0.674<br>p < 0.001	r = 0.839<br>p < 0.001	r = -0.300<br>p < 0.001	r = -0.343<br>p < 0.001	r = -0.166<br>p < 0.001
**Hb**	-	-	-	-	-	-	-	-	r = 0.806<br>p < 0.001	r = 0.201<br>p < 0.001	r = 0.215<br>p < 0.001	r = 0.088<br>p = 0.011
**Hct**	-	-	-	-	-	-	-	-	-	r = 0.245<br>p < 0.001	r = 0.134<br>p < 0.001	r = -0.168<br>p < 0.001
**MCV**	-	-	-	-	-	-	-	-	-	-	r = 0.889<br>p < 0.001	r = 0.026<br>p = 0.454
**MCH**	-	-	-	-	-	-	-	-	-	-	-	r = 0.472<br>p < 0.001
